# AglM and VNG1048G, Two Haloarchaeal UDP-Glucose Dehydrogenases, Show Different Salt-Related Behaviors

**DOI:** 10.3390/life6030031

**Published:** 2016-08-03

**Authors:** Lina Kandiba, Jerry Eichler

**Affiliations:** Department of Life Sciences, Ben Gurion University, Beersheva 84105, Israel; lina.kandiba@gmail.com

**Keywords:** Archaea, *Halobacterium salinarum*, *Haloferax volcanii*, protein glycosylation, UDP-glucose dehydrogenase

## Abstract

*Haloferax volcanii* AglM and *Halobacterium salinarum* VNG1048G are UDP-glucose dehydrogenases involved in *N*-glycosylation in each species. Despite sharing >60% sequence identity and the ability of VNG1048G to functionally replace AglM in vivo, these proteins behaved differently as salinity changed. Whereas AglM was active in 2–4 M NaCl, VNG1048G lost much of its activity when salinity dropped below 3 M NaCl. To understand the molecular basis of this phenomenon, each protein was examined by size exclusion chromatrography in 2 M NaCl. Whereas AglM appeared as a dodecamer, VNG1048G was essentially detected as a dodecamer and a dimer. The specific activity of the VNG1048G dodecamer was only a sixth of that of AglM, while the dimer was inactive. As such, not only was the oligomeric status of VNG1048G affected by lowered salinity, so was the behavior of the individual dodecamer subunits. Analyzing surface-exposed residues in homology models of the two UDP-glucose dehydrogenases revealed the more acidic and less basic VNG1048G surface, further explaining the greater salt-dependence of the *Hbt. salinarum* enzyme.

## 1. Introduction

It is now clear that life on Earth can thrive in environments once thought to be uninhabitable, including niches characterized by extremes of temperature, pH, or salinity [1,2]. As such, the proteins of organisms that exist in these demanding habitats must remain in a folded and functional state in the face of conditions that would normally lead to protein denaturation, loss of solubility, and aggregation. For example, whereas exposure to molar concentrations of salt compromises protein tertiary structure by increasing hydrophobic interactions within the folded polypeptide and interferes with electrostatic-based protein-protein interactions, hypersalinity does not have such effects on the proteins of highly halophilic organisms, defined as organisms found in surroundings containing >2 M NaCl [3,4,5,6]. 

To decipher the basis for such stability, numerous studies have compared the amino acid sequences and, when available, the structures of halophilic proteins and their non-halophilic counterparts. Such efforts have shown that halophilic protein sequences present an increased number of acidic residues (i.e., aspartic and glutamic acids) and reduced number of basic residues (lysines, in particular), relative to their non-halophilic counterparts [3,5,7,8]. Moreover, halophilic proteins contain lower hydrophobic residue contents than do their non-halophilic counterparts. Accordingly, analysis of the limited number of available halophilic protein structures has revealed that the surfaces of such proteins are highly negatively charged and present small hydrophobic patches, relative to their non-halophilic homologues [6,9,10,11,12,13,14]. A decrease in accessible surface area is also thought to contribute to protein halophilicity [6,14,15]. Still, the manner by which these and other observed molecular adaptations translate into the ability of halophilic proteins to overcome the challenges presented by hypersaline surroundings remains a matter of debate [4,6,7,10,15,16,17,18,19,20]. At the same time, the question of whether those molecular traits that distinguish a halophilic protein from its non-halophilic homologue also hold true when comparing homologous enzymes from halophiles that grow at different levels of hypersalinity or whether additional adaptations are involved remains largely unaddressed. 

Like many other Archaea, the halophiles *Haloferax volcanii* and *Halobacterium salinarum* both perform *N*-glycosylation, namely the covalent linkage of glycans to select asparagine residues of target proteins (for recent review, see [21]). In the case of *Hfx. volcanii*, two pathways of *N*-glycosylation have been deciphered [22,23], while the same may also hold true for *Hbt. salinarum* [24,25]. In the *Hfx. volcanii* Agl (*a*rchaeal *gl*ycosylation) pathway responsible for the assembly and attachment of an Asn-linked pentasaccharide, AglM acts as a UDP-glucose dehydrogenase, converting UDP-glucose into UDP-glucuronic acid [26]. In *Hbt. salinarum*, where glycoproteins are modified by an N-linked glycan of similar composition, *VNG1048G*, encoding a homologue of AglM, is not only found within a cluster of *N*-glycosylation-related genes reminiscent of the genomic region surrounding its *Hfx. volcanii* counterpart but can also functionally replace *aglM* in a *Hfx. volcanii* strain lacking this gene [25]. Yet, despite their functional similarities, AglM and VNG1048G are found in organisms that grow optimally at different salinities. Whereas *Hfx. volcanii* grows optimally in 1.7–2.5 M NaCl [27], *Hbt. salinarum* requires 3–5 M NaCl in the medium for proper growth and cell shape [28]. These inherent differences thus raise questions related to how AglM and VNG1048G behave as salinity changes.

In the present study, *Hfx. volcanii* AglM and *Hbt. salinarum* VNG1048G were compared in terms of the effects of decreased salinity on their enzymatic activity and their tertiary and quaternary structures. The findings reported here offer further insight into the molecular adaptations that allow halophilic proteins to properly fold and function in the presence of molar concentrations of salt.

## 2. Materials and Methods 

### 2.1. Strains and Growth Conditions

*Hfx. volcanii* WR536 (H53) cells were grown in medium containing 3.5 M NaCl, 0.16 M MgSO_4_, 1 mM MnCl_2_, 5 mM KCl, 3 mM CaCl_2_, 0.3% (*w*/*v*) yeast extract, 0.5% (*w*/*v*) tryptone, 50 mM Tris-HCl, pH 7.2 [29].

### 2.2. Plasmid Construction

To introduce a *Clostridium thermocellum* cellulose-binding domain (CBD) tag into *Hfx. volcanii* AglM and *Hbt. salinarum* VNG1048G, plasmid pWL-CBD-AglD [30] was linearized using *Nde*I and *Kpn*I, releasing the *aglD* sequence. Next, PCR amplification was performed using a forward primer spanning the 3’ end of the CBD-coding sequence and the 5’ end of *aglM* or VNG1048G, and a reverse primer spanning the 3’ end of each gene and the sequence downstream of the *Kpn*I site, together with *Hfx. volcanii* or *Hbt. salinarum* genomic DNA as template, respectively. The sequences of the primers used are listed in Table 1. Each amplified PCR fragment was ligated into the linearized pWL-CBD plasmid and the resulting plasmids, pWL-CBD-AglM, and pWL-CBD-VNG1048G, were individually introduced into *Hfx. volcanii* cells.

To generate plasmids encoding C-terminally polyhistidine-tagged *Hfx. volcanii* AglM and *Hbt. salinarum* VNG1048G, each gene was PCR amplified using primers designed to introduce *Nde*I and *Xho*I restriction sites at the start and end of each gene, respectively. The amplified fragments were inserted into the pET24 plasmid (Novogen) so as to introduce DNA encoding a polyhistidine tag, cleaved with the same enzymes. The tag-bearing fragment was then excised upon digestion with *Nde*I and *Blp*I and ligated into plasmid pJAM-202 [31], previously digested with the same restriction enzymes, to produce plasmids pHis-AglM and pHis-VNG1048G. The plasmids were then introduced into *Hfx. volcanii* cells.

### 2.3. Protein Purification

CBD-tagged *Hfx. volcanii* AglM and *Hbt. salinarum* VNG1048G were purified as previously described [32]. Briefly, 1 mL aliquots of *Hfx. volcanii* cells transformed to express CBD-AglM [26] or CBD-VNG1048G [25] were grown to mid-logarithmic phase, harvested and resuspended in 1 mL solubilization buffer (1% Triton X-100, 3 mg/mL DNaseI, 0.5 mg/mL PMSF, 50 mM Tris-HCl, pH 7.2) containing 2 M NaCl. The solubilized mixture was mixed for 20 min at 4 °C, after which time 50 mL of a 10% (*w*/*v*) solution of cellulose was added. After a 120 min nutation at 4 °C, the suspension was centrifuged (3000 *g* for 5 min), the supernatant was discarded and the cellulose pellet was washed four times with wash buffer containing 2 M NaCl, 50 mM Tris-HCl, pH 7.2. After the final wash, the cellulose beads were centrifuged (3000 *g* for 5 min), the supernatant was removed and the pellet, containing cellulose beads linked to the CBD-tagged proteins, was employed in an in vitro UDP-glucose dehydrogenase activity assay.

Polyhistidine-tagged *Hfx. volcanii* AglM and *Hbt. salinarum* VNG1048G were purified as previously described [33]. Briefly, 3 L cultures of each transformed strain were harvested and resuspended in 100 mL of buffer (20 mM imidazole, 2 M NaCl, 5 mM MgCl_2_, 50 mM Tris-HCl, pH 9.2). The cells were disrupted by sonication (35% output, four times for 30 s, 2 s on and 1 s off; Misonix XL2020 ultrasonicator, Farmington, NY, USA) on ice, and 1 ml (*v*/*v*) of 1% Triton X-100 was added. The lysate was subjected to ultracentrifugation (133,000 *g*, 1 h, 4 °C) and the supernatant (100 mL) was loaded onto a Ni-NTA (GE Healthcare, Little Chalfont, Buckinghamshire, UK) column (1 × 5 mL) attached to a BioRad BioLogic Duoflow 10 chromatorography system at a flow rate of 1 mL/min, previously equilibrated with 20 mM imidazole, 2 M NaCl, 5 mM MgCl_2_, 50 mM Tris-HCl, pH 9.2. Following washing with the equilibration buffer (15 mL), bound protein was eluted upon addition of a 10–250 mM imidazole gradient in 2 M NaCl, 5 mM MgCl_2_, 50 mM Tris-HCl, pH 9.2.

### 2.4. Size-Exclusion Chromatography

Directly following NiNTA-based chromatography, His-tagged AglM and VNG1048G were subjected to size-exclusion chromatography on a Hi-Load Superdex 200 16/60 cm (GE Healthcare, Little Chalfont, Buckinghamshire, UK) column equilibrated in buffer containing 2 M NaCl, 2 M NaCl, 5 mM MgCl_2_, 50 mM Tris-HCl, pH 9.2, using a BioRad BioLogic Duoflow 10 chromatography system at a flow rate of 1 mL/min. Molecular mass calibration markers (β-amylase, 200 kDa; alcohol dehydrogenase, 150 kDa; albumin, 66 kDa; carbonic anhydrase, 29 kDa and cytochrome c, 12.4 kDa) came from Sigma (St. Louis, MO, USA). Dextran blue was used to calculate the column void volume.

### 2.5. UDP-Glucose Dehydrogenase Activity Assay

The UDP-glucose dehydrogenase activities of CBD-AglM and CBD-VNG1048G were assayed in buffer containing 2–4 M NaCl, 5 mM MgCl_2_, 50 mM Tris-HCl, pH 9.2, as appropriate, essentially as described previously [26]. The UDP-glucose dehydrogenase activities of His-tagged AglM and His-tagged VNG1048G were assayed by incubating a 960 µL aliquot of size exclusion chromatography peak fractions in the absence or presence of 5 mM NAD^+^ (20 µL) and/or 2 mM UDP-glucose (20 µL) over 30 min. NADH concentration was calculated from a standard curve generated from absorbance at 340 nm in the presence of 0–1000 µM of NADH. Protein concentration was determined using Bradford reagent (BioRad, Hercules, CA, USA) and a bovine serum albumin-based calibration curve.

### 2.6. Bioinformatics

Sequences were aligned using the ClustalW server (https://www.clustal.org/clustal2). The Boxshade tool at the SDSC Biology WorkBench (http://workbench.sdsc.edu/) was used for presentation of the aligned sequences. pI values were calculated using Compute pI/Mw as found at the ExPasy bioinformatics resource portal (http://web.expasy.org/cgi-bin/compute_pi/pi_tool).

### 2.7. Homology Modeling

Homology models of *Hfx. volcanii* AglM and *Hbt. salinarum* VNG1048G were generated by Phyre2 (http://www.sbg.bio.ic.ac.uk/phyre2/html/page.cgi?id=index), using the default settings. The solved structures of *Streptococcus pyogenes* UDP-glucose dehydrogenase (PDB code 1DLI; [34]) and human UDP-glucose dehydrogenase (PDB code 2Q3E; [35]) were selected as templates for modeling. The homology models generated were superimposed on the solved UDP-glucose dehydrogenase structures by UCSF-Chimera (https://www.rbvi.ucsf.edu/chimera/index.html), using the default settings.

## 3. Results

### 3.1. Hfx. volcanii AglM and Hbt. salinarum VNG1048G Activities Are Differently Affected by Decreasing Salinity

**Figure 1:**
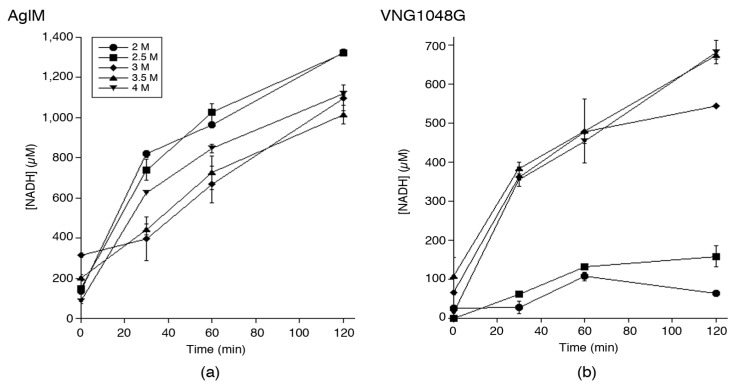
Salt-dependency of AglM and VNG1048G UDP-glucose dehydrogenase activity. The effects of increasing concentrations of NaCl on the UDP-glucose dehydrogenase activity of cellulose-bound CBD-AglM (**a**) and CBD-VNG1048G (**b**) were tested over the course of 120 min. Each value represents the average of three repeats ± standard deviation.

As a first step in characterizing *Hfx. volcanii* AglM and *Hbt. salinarum* VNG1048G, in vitro assays were conducted to assess the ability of each enzyme to catalyze the NAD^+^-dependent oxidation of UDP-glucose as a function of buffer salinity. Accordingly, each enzyme was engineered with a C-terminal *C. thermocellum* CBD tag that allows for cellulose-based purification in high salt surroundings [32] and expressed in *Hfx. volcanii* [25,26]. Cellulose-bound CBD-tagged AglM and VNG1048G were subsequently added to buffer containing 2, 2.5, 3, 3.5, or 4 M NaCl and the conversion of NAD^+^ to NADH was measured by spectrophotometrically determining absorbance at 340 nm over time. In this manner, distinct effects of salt concentration differences on the ability of the two enzymes to generate NADH were observed. *Hfx. volcanii* AglM generated similar amounts of NADH over time at each salt concentration tested, with the highest such activity been recorded in buffer containing 2 or 2.5 M NaCl. The amount of NADH generated by AglM did not substantially change as buffer salinity increased to 4 M NaCl (Figure 1a). By contrast, *Hbt. salinarum* VNG1048G showed the highest levels of NADH production in buffer containing 3.5 or 4 M NaCl. However, in buffer containing less than 3 M NaCl, only poor such VNG1048G activity was seen (Figure 1b). As such, whereas AglM is functional in the presence of 2–4 M NaCl, VNG1048G requires a milieu containing more than 2.5 M NaCl to display comparable activity. 

### 3.2. In 2 M NaCl, AglM, and VNG1048G Differ in Terms of Oligomeric Status 

On the basis of protein size and signature sequence motifs, UDP-glucose dehydrogenases like AglM and VNG1048G can be assigned to one of two groups, with that version of the enzyme found in eukaryotes being distinguished from the same enzyme derived from essentially bacterial and archaeal sources [36]. Further support for this division comes from analysis of the solved crystal structures of *Caenorhabditis elegans* (PDB code 2O3J) and *S. pyogenes* (PDB code 1DLI and 1DLJ; [34]) UDP-glucose dehydrogenases, corresponding to representatives of each group, respectively. Whereas the eukaryotic version of the enzyme exists as a hexamer, the bacterial enzyme is found as a dimer. The only archaeal UDP-glucose dehydrogenase structure solved to date, namely that of the hyperthermophile *Pyrobaculum islandicus*, also exists as a dimer [37]. With this in mind, efforts were undertaken to define the quaternary structures of *Hfx. volcanii* AglM and *Hbt. salinarum* VNG1048G and to determine whether both enzymes present the same oligomeric status at low salt levels. 

**Figure 2:**
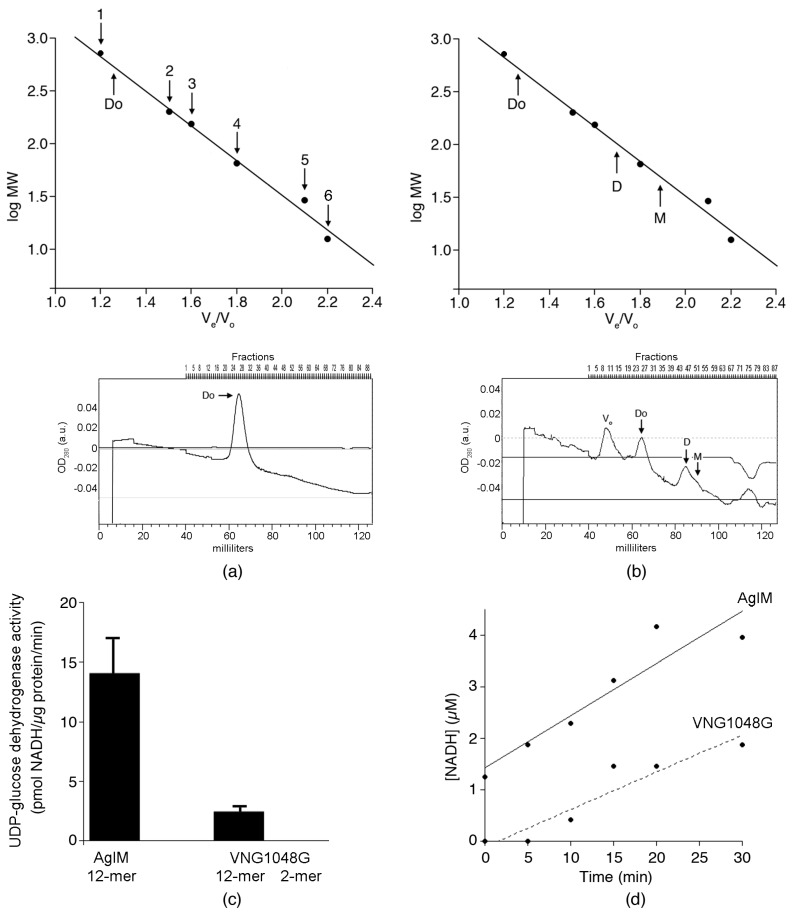
In 2 M NaCl, AglM, and VNG1048G present different quaternary structures. NiNTA-purified AglM (**a**) and VNG1048G (**b**) were subjected to size exclusion chromatography (see Materials and Methods). The upper half of (**a**) and (**b**) shows a calibration curve generated based on the elution of β-amylase (1; 200 kDa), alcohol dehydrogenase (2; 150 kDa), bovine serum albumin (3; 66 kDa), carbonic anhydrase (4; 29 kDa), and cytochrome c (5; 12.4 kDa). Dextran blue (2000 kDa) was used to determine the void volume (V_o_). The calibration curve served to determine the molecular masses of peaks determined as representing dodecamers (Do), dimers (D), and monomers (M) of AglM or VNG1048G, as indicted. In (**a**), the lower half of the panel presents the AglM elution profile, while in (**b**), the lower half of the panel presents the VNG1048G elution profile. In both profiles, the peaks corresponding to the different oligomeric states of each enzyme are indicated, whereas the identities of the points on the calibration curves are added in (**a**). (**c**) The activities of the AglM dodecamer, and the VNG1048G dodecamer and dimer are shown normalized per µg protein per minute (over the course of a 30 min assay). The values portrayed represent the average of two repeats of the experiment ± standard deviation. (**d**) The activities of the AglM (full line) and VNG1048G (dashed line) dodecamers in a representative experiment. Line fitting (*R* = 0.93 for AglM and *R* = 0.94 for VNG1048G) was achieved using KaleidaGraph v.4.1 (Synergy Software, Reading, PA, USA)

Plasmids encoding His-tagged versions of AglM and VNG1048G were introduced into *Hfx. volcanii* cells. Subsequently, each His-tagged protein was purified on NiNTA resin in buffer containing 2 M NaCl (both eluting in 100 mM imidazole) and subjected to size-exclusion chromatography in buffer containing the same concentration of salt. Because all attempts to store the purified His-tagged enzymes for subsequent examination proved unsuccessful, size exclusion chromatography was instead performed immediately after enzyme purification on NiTNA resin. Such analysis revealed that AglM eluted as a single peak corresponding to a molecular mass of 550 kDa (Figure 2a). In contrast, the elution profile of VNG1048G contained two major peaks corresponding to 550 and 99 kDa species, a smaller peak corresponding to a 47 kDa species and two very minor peaks corresponding to 280 and 191 kDa species (Figure 2b). Based on their amino acid sequences, the calculated molecular masses of His-tagged AglM and VNG1048G are close to 47 kDa. As such, it would appear that the 550 kDa species seen in both the AglM and the VNG1048G elution profiles corresponds to a dodecamer, whereas the 99 and 47 kDa species in the VNG1048G elution profile are a dimer and monomer, respectively. Moreover, the minor 280 and 191 kDa species noted in the VNG1048G profile likely correspond to hexamers and tetramers, respectively. Coomassie staining of the fractions comprising each of the peaks that appeared in both elution profiles as listed above following SDS-PAGE confirmed the presence of UDP-glucose dehydrogenase in each case (not shown). 

Finally, it was considered whether the fractions comprising the eluted AglM- and major VNG1048G-containing peaks displayed UDP-glucose dehydrogenase activity. Accordingly, enzyme-containing fractions were assayed over a 30 min period. Such analysis revealed that the specific activity of the AglM dodecamer greatly exceeded that of the VNG1048G dodecamer (14.3 ± 2.7 versus 2.5 ± 0.4 pmol NADH/µg protein/min, respectively). In contrast, no activity was detected for the VNG1048G dimer (Figure 2c). In more closely examining the accumulation of NADH over time in a representative experiment (Figure 2d), the initial rates of AglM (101.2 *n*M NADH/min) and NADH (72.1 *n*M NADH/min) activities are shown. The linear increase in NADH accumulation shows that both dodecamers remained stably over the course of the assay. It would thus appear that the loss of VNG1048G activity seen at the lower salinities tested is due both to changes in the oligomeric status of VNG1048G and to changes associated with the individual VNG1048G subunits of the active dodecamer. 

### 3.3. VNG1048G Contains More Acidic and Fewer Basic Residues than AglM

In seeking explanations for the observed differences in AglM and VNG1048G enzymatic activity as a function of salinity apart from oligomeric status, the amino acid contents of the two halophilic UDP-glucose dehydrogenases were considered. Both *Hfx. volcanii* AglM (430 residues) and *Hbt. salinarum* VNG1048G (426 residues) are enriched in acidic (Asp, Glu) and poor in basic (Arg, Lys) residues, relative to human UDP-glucose dehydrogenase isoform 1 (494 residues), an example of a non-halophilic homologue. Specifically, AglM, VNG1048G, and the human enzyme contain 77 (18%), 80 (19%), and 63 (13%) acidic residues and 40 (9%), 31 (7%), and 62 (13%) basic residues, respectively. The enhanced acidic characters of AglM and VNG1048G are also reflected by their pI values (4.31 and 4.17, respectively), relative to that of the human enzyme (6.73). As such, the amino acid contents of AglM and VNG1048G are typical of halophilic proteins. 

**Figure 3:**
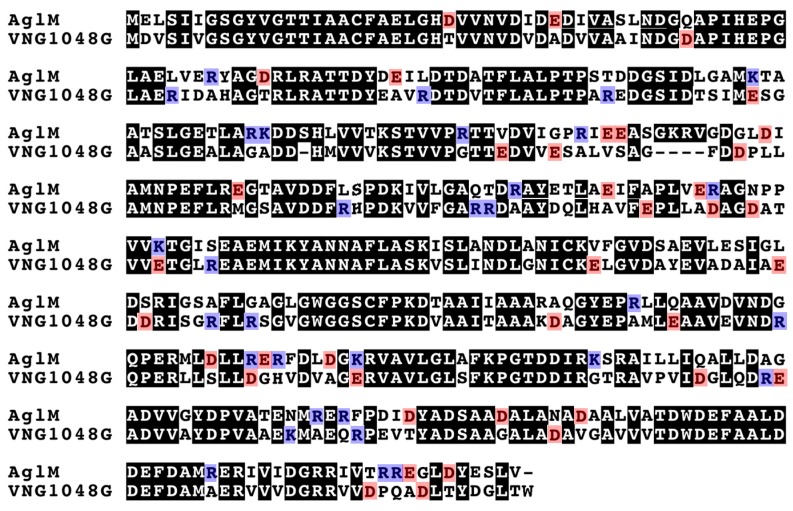
Sequence alignment of *Hfx. volcanii* AglM and *Hbt. salinarum* VNG1048G. Identical residues shared by AglM and VNG1048G are shown in white against a black background. Residues that differ between AglM and VNG1048G are shown, apart from those positions where similar residues (D/E, K/R, L/V/I/M, S/T, A/G) are seen. Acidic residues (D,E) are shown against a red background, while basic residues (K,R) are shown against a blue background.

The amino acid sequences of *Hbt. salinarum* VNG1048G and *Hfx. volcanii* AglM were next compared to each other. Sequence alignment revealed that AglM and VNG1048G share 64% sequence identity over their entire lengths (Figure 3). Of the remaining residues, 30% showed substantial similarity, with Asp or Glu, Lys or Arg, Leu, Ile. Val or Met, Ser or Thr, or Ala or Gly being found at the corresponding positions in each protein. In the remaining 24% of the AglM and VNG1048G sequences, termed the protein-specific amino acid pool of each protein, it was noted that AglM presents 18 acidic and 21 basic residues, while VNG1048G presents 21 acidic and 13 basic residues. In other words, VNG1048G contains more acidic and fewer basic residues than does AglM. Further examination revealed that VNG1048G also presents more acidic residues at position occupied by basic residues in AglM than vice versa. 

### 3.4. More Acidic and Fewer Basic Residues are Surface-Exposed in VNG1048G Than in AglM 

**Figure 4:**
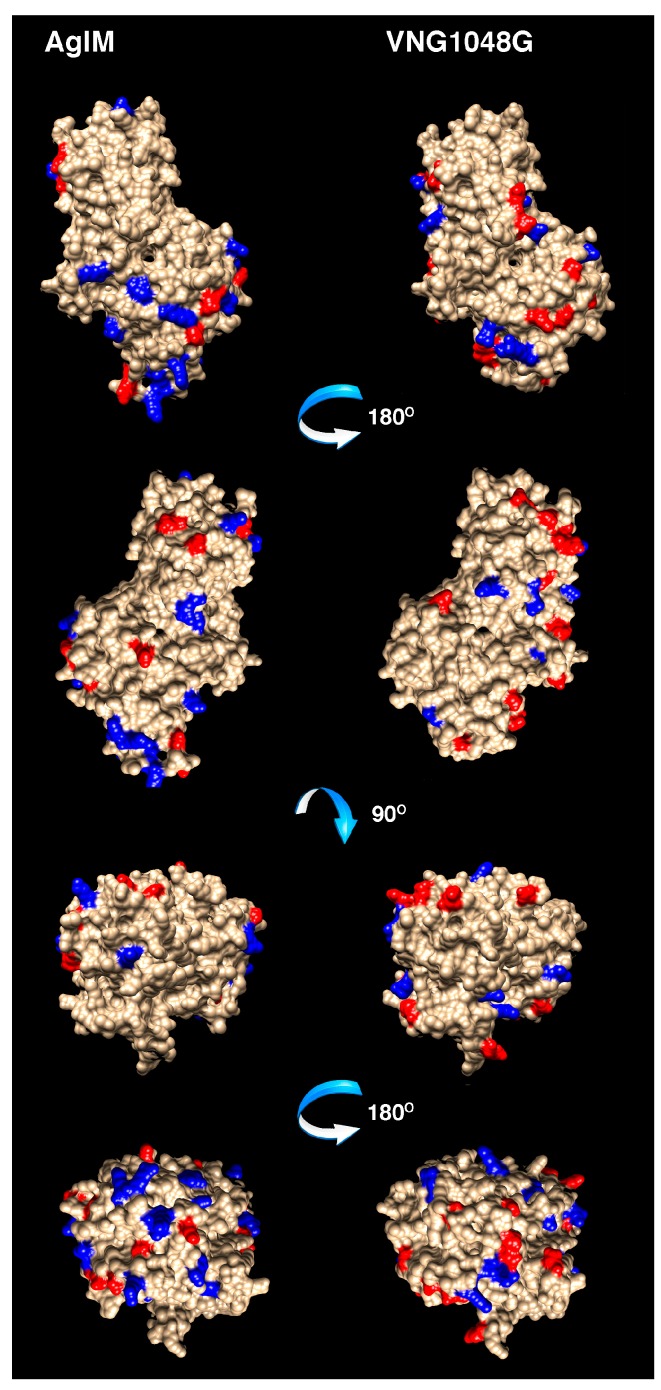
Homology models of AglM and VNG1048G. Homology models of AglM (**left**) and VNG1048G (**right**) were generated based on the solved crystal structure of human UDP-glucose dehydrogenase (PDB code 2Q3E). Shown are the front (top panels), back (panels second from top), top (panels third from top) and bottom (bottom panels) views of both models. Protein-specific acidic residues are colored in red, while protein-specific basic residues are colored in blue.

To better visualize differences that distinguish AglM from VNG1048G, the three-dimensional structures of AglM and VNG1048G were considered. However, as no structural data have been reported for either protein, homology models were generated for each using the Phyre2 protein fold recognition server, with the crystal structures of the human (PDB code 2Q3E) (Figure 4) and the *S. pyogenes* (PDB code 1DLI) enzymes (not shown) as templates. The hexameric human UDP-glucose dehydrogenase structure [35,38] was selected as a template since AglM and VNG1048G exist as dodecamers (Figure 2), possibly corresponding to paired hexamers. However, as AglM and VNG1048G contain sequence motifs and are of lengths that group these enzymes with other prokaryotic UDP-glucose dehydrogenases [36], the structure of the *S. pyogenes* enzyme was also used as template.

The reliability of the models generated was reflected in the low root mean square deviations (RMSDs) obtained. For the AglM model based on human structure (30% sequence identity), an RMSD value of 0.246 Å was measured, while for the AglM model based on the *S. pyogenes* structure (25% sequence identity), an RMSD of 0.232 Å was determined. An RMSD of 0.213 Å was reported for the VNG1048G model based on the human UDP-glucose dehydrogenase structure (27% sequence identity), whereas an RMSD of 0.271 Å was obtained with the *S. pyogenes* structure (22% sequence identity) serving as template. Moreover, the RMSD of the AglM model from the VNG1048G model was 0.277 Å when both were based on the eukaryal structure, and 0.235 Å when both were based on the bacterial structure. These values reflect the overall similarity of the predicted haloarchaeal enzyme structures. Finally, the area of solvent accessible surface of each model was 22,005 Å^2^ for AglM and 21,318 Å^2^ for VNG1048G when the models were based on the human structure and 22,096 Å^2^ for AglM and 20,124 Å^2^ for VNG1048G when the models were based on the *S. pyogenes* structure. The decreased area of solvent accessible surface of the more halophilic *Hbt. salinarum* protein is in agreement with earlier studies reporting that such a decrease underlies the ability of halophilic proteins to tolerate hypersalinity [6,15]. 

When the positions of the AglM and VNG1048G protein-specific acidic and basic residues were addressed in the models generated based on the human structure, 13 acidic and 17 basic residues were assigned to the surface of AglM, as opposed to 17 acidic and 12 basic residues for VNG1048G (Figure 4). When the same residue sets were assessed in the AglM and VNG1048G models based on the *S. pyogenes* UDP-glucose dehydrogenase structure, 12 acidic and 18 basic residues were assigned to the AglM surface, while 18 acidic and 8 basic residues were assigned to the VNG1048G surface (not shown). Thus, not only does VNG1048G contain more acidic and fewer basic residues than does AglM, VNG1048G exposes more acidic and fewer basic residues on the protein surface than does AglM.

## 4. Discussion

Deciphering how proteins remain folded and functional in the face of harsh physical conditions carries implications not only for better understanding of life at extremes but also for designing proteins optimized to act in particular surroundings for applied purposes. In the present study, explanations for the distinct salt requirements of *Hfx. volcanii* AglM and *Hbt. salinarum* VNG1048G, two halophilic UDP-glucose dehydrogenases, were sought. While numerous studies have compared halophilic proteins with their non-halophilic counterparts in an attempt to understand how the former cope with hypersalinity [3,5,6,17], this report is, to the best of our knowledge, the first to directly address the effects of salinity on the activity of the same enzyme from two widely-used haloarchaeal model systems, namely *Hfx. volcanii* and *Hbt. salinarum* [39,40]. 

In attempting to define the molecular basis for the distinct salinity requirements of AglM and VNG1048G, the oligomeric status of each enzyme was considered. It was initially assumed that these enzymes exist as dimers, as do other prokaryotic UDP-glucose dehydrogenases [34,41], including *P. islandicus* UDP-glucose dehydrogenase, the only archaeal version of the enzyme for which structural information is available [37,42]. By contrast, eukaryotic versions of the enzyme exist as hexamers, apparently formed from the trimerization of dimers [38,43,44,45]. Size exclusion chromatography performed in 2 M NaCl, however, revealed AglM to be a dodecamer. A dodecameric population of VNG1048G was also discerned in 2 M NaCl, along with a major pool of dimers, some monomers, and minor amounts of hexamers and tetramers. The possibility that the AglM and VNG1048G dodecamers correspond to pairs of interacting hexamers is supported by the observation that the VNG1048G size exclusion chromatography profile included dodecamers and hexamers but no species intermediate to these groups. Moreover, the detection of VNG1048G monomers, dimers, and multiples thereof (but not trimers) could reflect the dimeric form of the enzyme serving as the basic building block of higher ordered multimers. 

In contrast to the functional VNG1048G dodecamers, no enzymatic activity could be measured for dimers of the enzyme. Indeed, links between salinity and the oligomeric status and activity of halophilic enzymes have been previously reported. Active *Haloarcula marismortui* malate dehydrogenase tetramers dissociates into inactive monomers at low salinity [46]. *Haloarcula sinaiiensis* nucleoside diphosphate kinase is found as an active hexamer at salt concentrations above 2 M but is converted into a less active dimer when salt levels drop below 1 M [47]. Similarly, tetrameric *Hfx. volcanii* alcohol dehydrogenase dissociates into less active dimers as salinity drops [48]. At the same time, a comparison of the activities of AglM and VNG1048G dodecamers normalized per amount of protein revealed AglM to be almost six-fold more active. It would thus appear that VNG1048G not only underwent changes in conformation at lower salinity that led to dodecamer disassembly but also that the individual VNG1048G subunits comprising the dodecamer underwent low salt-induced changes that compromised activity. 

As part of a strategy to remain properly folded and functional in hypersaline surroundings, halophilic protein surfaces are enriched in acidic residues, relative to their non-halophilic homologues [3,5]. Consequently, halophilic proteins unfold upon encountering low salt surroundings [4,9,13]. In the present study, homology modeling attributed the increased acidity and decreased basicity of VNG1048G, relative to AglM, to residues localized to the protein surface. It is, therefore, likely that upon exposure to 2 M NaCl, VNG1048G lost its proper folding, leading to a decrease in catalytic activity, again, relative to AglM. As a result, VNG1048G surface residues normally shielded at higher salt levels could be exposed at the lower salinities, leading to destabilization of the VNG1048G dodecamer and the appearance of the lower-ordered multimers seen. With a less negatively charged surface, AglM could avoid this situation and maintain its dodecameric status. Still, it is likely that additional factors contribute to the enhanced halophilicity of VNG1048G. For instance, site-directed mutation studies support the concept that adaption of halophilic enzymes to highly saline surroundings is not only due to increased acidic residue content but also due to the structural distribution and organization of such residues [15,20,49,50,51,52]. Indeed, very subtle changes may lead to substantial differences in halophilicity. Differing by only a single residue, *Haloarcula quadrata* and *Har. sinaiiensis* nucleoside diphosphate kinases are optimally active at 1 and 2 M NaCl, respectively [47].

In summary, the present study argues that both oligomeric considerations and folding of the individual subunits in such oligomers contributes to the higher salt concentrations required for *Hbt. salinarum* VNG1048G activity, as compared to its *Hfx. volcanii* homologue AglM. However, because different halophilic proteins rely on distinct inter- and intra-molecular considerations to realize proper folding and optimal activity, the generality of the findings reported here remains to be determined. It is also reasonable to assume that components present in the haloarchaeal cytoplasm could contribute to the stability of haloarchaeal proteins. Indeed, the conditions tested here do not reflect those of the cytoplasm of haloarchaea, which is thought to contain 4–5 M KCl [53,54]. Future study of halophilic proteins in their native surroundings will elucidate these and other points related to the relation between polypeptide folding, protein stability, and hypersalinity.

## Figures and Tables

**Table 1:** Primers used in this study.

Primer	Description	Sequence ^1^
VNG1048G NdeI	Forward primer introducing an *Nde*I site at the start of *VNG1048G* for cloning into plasmids pWL-CBD and pET24b	ccccatatgGACGTGAGCATCGTTGGGAGTGGG
VNG1048G StuI rev	Reverse primer introducing a *Stu*I site at the end of *VNG1048G* for cloning into plasmid pWL-CBD	gggaggcctCTACCAGGTGAGCCCGTCGTAGGTC
VNG1048G XhoI rev	Reverse primer introducing a *Xho*I site at the end of *VNG1048G* for cloning into plasmid pET24b	gggctcgagCCAGGTGAGCCCGTCGTAGGTCAGG
aglM NdeI	Forward primer introducing an *Nde*I site at the start of *aglM* for cloning into plasmids pWL-CBD and pET24b	gggcatatgGAACTCAGTATCATCGGGAG
aglM KpnI rev	Reverse primer introducing a *Kpn*I site at the end of *aglM* for cloning into plasmid pWL-CBD	cccggtaccTCAGACGAGCGACTCGTAGTCG
aglM XhoI rev	Reverse primer introducing a XhoI site at the end of *aglM* for cloning into plasmid pET24b	cccggtaccGACGAGCGACTCGTAGTCGAG

^1^ The introduced restriction site is given in lower case letters.
